# Knowledge of obstetric danger signs and associated factors among mothers in Bahir Dar district, northwest Ethiopia: an institution-based cross-sectional study

**DOI:** 10.1186/s40985-020-00132-7

**Published:** 2020-07-01

**Authors:** Merina Jewaro, Hedija Yenus, Yohanes Ayanaw, Birhanu Abera, Terefe Derso

**Affiliations:** 1 Department of Gynecology and Obstetrics, Government Public Health Facility, Kersa District Hospital, Arsi, Ethiopia; 2 Department of Reproductive Health, Institute of Public health, College of Medicine and Health Sciences, University of Gondar, Gondar, Ethiopia; 3 Department of Gynecology and Obstetrics, College of Medicine and Health Sciences, University of Gondar, Gondar, Ethiopia; 4 Department of Human Nutrition, Institute of Public Health, College of Medicine and Health Sciences, University of Gondar, P.O. Box 196, Gondar, Ethiopia

**Keywords:** Obstetric danger signs, Associated factors, Northwest Ethiopia

## Abstract

**Background:**

Like other developing countries, in Ethiopia, obstetric complications contribute to about 50% of the maternal deaths. Thus, the aim of this study was to assess knowledge of obstetric danger signs and its associated factors among mothers attending the postnatal clinic at Felege Hiwot Referral Hospital, Bahir Dar district, northwest Ethiopia.

**Methods:**

A hospital-based cross-sectional study was conducted on 410 postnatal mothers at Felege Hiwot Referral Hospital from June to September 2015. Knowledge of obstetric danger signs among postnatal clinic attending mothers was determined by using seventeen obstetric danger sign questions via interviewing. The binary logistic regression model was used to identify associated factors. In the multivariable analysis, variables with a *P* value of < 0.05 were considered statistically significant. Odds ratio with 95% confidence interval (CI) was calculated to show the strength of association.

**Results:**

About 59% [95% CI 55, 63] of mothers were knowledgeable about obstetric danger signs. The odds of having good knowledge of obstetric danger signs were higher among mothers who were more educated [AOR = 6.86, 95% CI 2.47, 19.27], earned more than 3500 ETB household monthly income [AOR = 3.38, 95% CI 1.20, 13.96], and received information on danger signs from health extension workers (HEWs) [AOR = 4.23, 95% CI 1.83, 9.70] compared to their counterparts. However, mothers with service utilization decision power [AOR = 0.14, 95% CI 0.07, 0.27] with husband were 86% times less likely to be knowledgeable compared to mothers decided by themselves.

**Conclusion:**

In this study, below two thirds of mothers had good knowledge of obstetric danger signs. Thus, improving educational status and obtaining health information on obstetric danger signs from health professionals should be intensified. This implies that lack of awareness may lead to delay in seeking care. Thus, improving mothers’ socio-economic status and self-decision-making power on utilization of health service are essential to mitigate the high burden of maternal morbidity due to obstetric complications. Also, obtaining health information on obstetric danger signs from health professionals should be intensified.

## Background

Obstetric danger signs are problems that mothers face during pregnancy, labor, and the postpartum period [1]. Globally, in 2013 alone, approximately 289,000 women died from complications related to pregnancy and childbirth, showing a decline by 45% from 1990 report [2, 3]. Ninety-nine percent of these deaths occurred in developing countries, especially in sub-Saharan Africa [4]. Like other developing countries, obstetric complications in Ethiopia contribute about 50% of the maternal deaths [5]. Although a marked decline of the burden of morbidity and mortality has been documented, 676 mothers die per 100,000 live births in Ethiopia. This huge proportion of maternal death is associated with obstetric hemorrhage and obstructed labor, mostly during or just after delivery [6, 7]. Sepsis, preeclampsia, eclampsia, and unsafe abortions are other direct obstetric complications during pregnancy, childbirth, or postpartum [1, 7].

Globally, different strategies support the implementation of skilled care by increasing women’s knowledge of obstetric danger signs and birth preparedness to mitigate maternal morbidity and mortality [8–10]. However, obstetric knowledge of danger signs remains low from studies conducted in Somali Region, Arba Minch town, East Gojjam, Raya Kobo, and Tsegedie district (15.5, 24.1, 55.1, 58.8 and 52.3%, respectively) among women of reproductive age in Ethiopia [11–15]. Studies conducted on the danger signs of obstetric complications in various settings identified several risk factors associated with good knowledge of the signs. The risk factors include marriage [16], old age [16, 17], antenatal care (ANC) follow-up [11, 15, 17], advanced education [13, 15–17], employment [15, 17], high parity [13, 17], institutional delivery [13, 15], urban residence [11, 13], good income [16], and decision-making power [16].

Over the last decade, Ethiopia has been implementing a reproductive strategy and recommended care during pregnancy (such as antenatal care, immunization, HIV testing for prevention of mother-to-child transmission of HIV (PMTCT) and nutrition intervention like iron-folic acid supplementation of free of charge) to reduce maternal death [18], though the magnitude of the problem still remains a public health problem and an unfinished agenda [9, 19]. Therefore, investigating knowledge of obstetric danger signs and associated factors is of paramount importance. However, women’s awareness about obstetric danger signs has substantial importance for improving maternal and child health; little is known about the current knowledge and influencing factors in the study area. Thus, this study aimed to assess knowledge of obstetric danger signs and its associated factors among mothers attending the postnatal clinic at Felege Hiwot Referral Hospital, Bahir Dar district, northwest Ethiopia, 2015.

## Methods

### Study design and setting

A hospital-based cross-sectional study was conducted from June to September, 2015, at Felege Hiwot Referral Hospital (FHRH). The hospital is found in Bahir Dar town, the capital of the Amhara Regional State, 567 km from Addis Ababa, the capital of Ethiopia. A total of 221,991 people live in the town [20]. The hospital is serving about 5 million inhabitants of Bahir Dar and the nearby catchment areas, such as West Gojjam, Awi, South Gondar, and parts of East Gojjam zones.

### Sampling procedures

All postnatal mothers attending the clinic within 6 weeks after delivery at the time of data collection period, irrespective of the birth outcome, were included in the study and considered as a study population. The sample size was calculated using the formula for estimating single population proportion by considering a 55.1% of expected prevalence of good knowledge of mothers on obstetric danger signs [13], a 95% level of confidence, a 5% margin of error, and a 10% non-response rate. Thus, the final sample size of 418 postnatal women would participate in the study. Mothers were selected from the postnatal clinic, using the systematic sampling technique based on their daily sequence of postnatal care registration, after identifying an initial starting mother by using a random number.

### Data collection procedure

A structured questionnaire was used to obtain knowledge of obstetric danger signs, socio-demographic information, and obstetric characteristics of the participants. First, the questionnaire was prepared in English and translated to Amharic, and then retranslated to English by professional translators and public health experts to check consistency. Finally, the Amharic version was administered. Three BSc degree graduate midwives collected the data, while another senior midwife supervised the process. One day training was given on the objective of the study, confidentiality of information, and techniques of an interview to data collectors and the supervisor. A pre-test was conducted on 5% (21) of the sample size out of the study area. During pre-test, the questionnaire was assessed for its clarity, wording, and the optimal time for completing the interview. Modifications were done based on the result. Investigators and supervisors regularly monitored the performance. All the collected questionnaires were checked daily for completeness by the supervisor and the primary investigator.

### Study variables and operational definitions

The outcome variable of the study was knowledge of obstetric danger signs. Thus, knowledge was determined based on ability to mention obstetric danger signs during pregnancy, childbirth, and the postpartum period. Knowledge of obstetric danger signs was determined by using seventeen obstetric complication questions. The questions were adapted from a safe motherhood questionnaire developed by the Maternal Neonatal Program of JHPIEGO [1]. In accordance with JHPIEGO definitions, knowledge was categorized into two categories, good and poor knowledge. Good knowledge means if the mother could have mentioned at least nine key obstetric danger signs during the three periods (pregnancy, delivery, and postpartum); otherwise, she is considered as having poor knowledge. The independent variables included in the study were socio-demographic such as age, maternal educational status, occupational status, marital status, family monthly income, and decision-making power in the house, and obstetric characteristics like gravidity, parity, antenatal care (ANC) visit, number of ANC visits, and mode of delivery. Decision-making power on service utilization was determined by asking mothers to provide information regarding who was the decision maker to use maternal health services (ANC, delivery, and postnatal services). Also, danger signs’ information from HEWs was measured asking mothers to provide information regarding were they informed about danger signs of obstetric complications during pregnancy, childbirth, and postpartum period by HEWs

### Data processing and analysis

Binary logistic regression models were used to identify variables which had associations with the dependent variable. Variables found to have a *p* value of up to 0.2 in the bivariable analysis, entered into the multivariable logistic regressions for controlling possible effects of confounders, and finally, the variables which had significant associations were identified on the basis of OR with 95% CI. In the multivariable analysis, variables with a *P* value of < 0.05 were considered statistically significant. Data were entered into the Epi Info version 3.5.1 and then exported to SPSS version 20.0 for analysis.

## Results

A total of 410 mothers (with a response rate of 98.1%) participated in the study. The mean age (± SD) of mothers was 27 (± 5.3) years with 349 (85.1%) mothers in the age group of 20–34 years. However, more than one third (35.4%) of mothers had no formal education; the majority (79.0%) were urban dwellers, and 95.6% were married. About 52.6% of the mothers had a history of one pregnancy, and of all participants, 90% have had antenatal care (ANC) visits. The vast majority (82.4%) of mothers gave birth in health institutions. More than two thirds (70.7%) of mothers had information on obstetric danger signs from health extension workers (HEWs) (Table 1).

**Table 1:** Socio-demographic and obstetric characteristics of mothers attending postnatal clinic at Felege Hiwot Referral Hospital, Bahir Dar, northwest Ethiopia, 2015 (*N* = 410)

Characteristics	Frequency	Percentage
**Age (in years)**		
18–19 years	44	10.7
20–34	349	85.1
35–47	17	4.2
**Religion**		
Orthodox	296	72.2
Muslim	55	13.4
Protestant	59	14.4
**Marital status**		
Married	392	95.6
Single	18	4.4
**Educational status**		
No formal education	145	35.4
Primary	104	25.4
Secondary	83	20.2
Above secondary	78	19.0
**Occupation of mothers**		
Housewife	79	19.3
Government employee	120	29.3
Farmer	163	39.8
Merchant	48	11.6
**Residence**		
Urban	324	79.0
Rural	86	21.0
**Educational status of husband (missing value = 18)**		
No formal education	102	26.0
Primary	120	30.6
Secondary	62	15.8
Above secondary	108	27.6
**Decision-making power on service utilization**		
Self	173	42.2
With husband	220	53.7
Another person	17	4.1
**Monthly income**		
ETB < 1200	111	27.1
ETB 1201–2000	118	28.8
ETB 2001–3500	88	21.5
ETB > 3500	93	22.6
**Time to reach to the hospital**		
< 30 min	324	79%
> 30 min	86	21%
**Number of pregnancy**		
1	216	52.6
2–4	147	35.9
5+	47	11.5
**Parity**		
1	216	52.6
2–4	147	35.9
5+	47	11.5
**Mode of delivery**		
Spontaneous vaginal delivery (SVD)	285	69.5
Cesarean section(C/S)	102	24.9
Instrumental delivery	23	5.6
**Fetal outcome**		
Live birth	347	84.6
Abnormal^$^	55	13.4
Dead	8	2.0
**Gestation at ANC visit started**		
< 4 months	126	30.7
4–7 months	187	45.6
> 8 months	97	23.7
**Number of ANC visit (missing value = 40)**		
1	41	11.1
2	98	26.5
3	86	23.2
4+	145	39.2
**Place of birth**		
Home	72	17.6
Health institution	338	82.4
**Danger signs information from HEW**		
Yes	290	70.7
No	120	29.3

*ETB* Ethiopia birr

^$^Low birth weight, preterm birth, IUGR, and congenital abnormality

About 59% [95% CI 55, 63] of mothers had good knowledge of obstetric danger signs. The participants reported 80.0% [95% CI 76.1, 83.9], 78.1% [74.2, 82.1], and 73.7% [69.5, 78.0] of vaginal bleeding, premature rupture of membrane, and increased/decreased fetal movement as the most frequent obstetric danger signs, respectively (Table 2).

**Table 2:** Knowledge of obstetric danger signs among mothers attending postnatal clinic at Felege Hiwot Referral Hospital, Bahir Dar, northwest Ethiopia, 2015

Obstetric danger signs	Proportion of mentioned danger signs	
	Yes (%)	No (%)
Vaginal bleeding	328(80.0)	82(20.0)
Severe headache	273(66.6)	137(33.4)
Convulsion	263(64.1)	147(35.9)
Loss of consciousness	286(69.8)	124(30.2)
Severe abdominal pain	253(61.7)	157(38.3)
Blurred vision	196(47.8)	214(52.2)
Increased/decreased fetal movement	302(73.7)	108(26.3)
Difficulty in breathing	189(46.1)	221(53.9)
Excessive vomiting	201(49.1)	209(50.9)
Preterm labor (onset of labor before 37 weeks of gestation)	284(69.3)	126(30.7)
Premature rupture of membrane/water breaks without labor	308(75.1)	102(24.9)
Prolonged labor(lasting > 12 h)	312(76.1)	98(23.9)
Retained placenta	287(70.0)	123(30.0)
Offensive vaginal discharge	197(48.0)	213(52.0)
High fever	214(52.2)	196(47.8)
Swollen hands/feet	73(17.8)	337(82.2)
Severe weakness	108(26.3)	302(73.7)

### Factors associated with knowledge on obstetric danger signs

In the bivariable analysis, most of the variables were found associated with a *p* value of less than 0.2. However, the result of the multivariable analysis revealed a *p* value of less than 0.05 that the odds of having good knowledge on obstetric danger signs were 6.86 times higher among mothers who had above secondary school education [AOR = 6.86, 95% CI 2.47,19.27] compared to mothers who had no formal education. Besides, mothers who had more than 3500 ETB household monthly income [AOR = 3.38, 95% CI 1.20, 13.96] were found to be more likely to have good knowledge compared mothers who had less than 1200 ETB household income. Mothers who received health information [AOR = 4.23, 95% CI 1.83, 9.70] on obstetric danger signs from HEWs were 4.23-fold more likely to be knowledgeable than mothers who received no such information. However, mothers who decided on service utilization with their husbands were 86% times less likely to be knowledgeable compared to mothers who decided by themselves [AOR = 0.14, 95% CI 0.07, 0.27] (Table 3).

**Table 3:** Factors associated with knowledge of obstetric danger signs at Felege Hiwot Referral Hospital, Bahir Dar district, northwest Ethiopia, 2015

Variables	Knowledge		COR (95% CI)	AOR (95% CI)
	Good	Poor		
**Residence**				
Rural	97(48.4)	102(51.6)	2.31(1.54, 3.45)	1.10(0.36, 3.40)
Urban	145(68.7)	66(31.3)	1.00	1.00
**Household monthly income**				
< 1200	43(38.7)	68(61.3)	1.00	1.00
1200–2000	55(46.6)	63(53.4)	7.61 (3.93, 14.72)	4.08(0.87, 19.16)
2001–3500	67(76.1)	21(23.9)	5.51 (2.88, 10.54)	6.55(0.99, 35.63)
> 3500	77(82.8)	16(17.2)	2.50(1.72, 3.12)	3.38(1.20, 13.96)*
**Mothers’ education**				
No formal education	59(40.7)	86(59.3)	1.00	1.00
Primary	66(63.5)	38(36.5)	4.85(2.60, 9.05)	2.91(0.47, 19.27)
Secondary	57(68.7)	26(31.3)	1.91 (0.99, 3.71)	4.94(0.68, 5.57)
Above secondary	60(76.9)	18(23.1)	1.52(0.75, 3.06)	6.86(1.62, 19.27)*
**Decision-making power on service utilization**				
Self	62(35.8)	111(64.2)	1.00	1.00
With husband	174(79.1)	46(20.9)	7.24(0.083–0.208)	0.14(0.07, 0.27)*
Others^#^	6(35.3)	11(64.7)	1.19(0.927–18.92	0.29(0.04, 2.06)
**ANC visit**				
No	12(23.1)	40(66.9)	5.98(10.23, 54.18)	7.24(0.50, 10.64)
Yes	230(64.2)	128(35.8)	1.00	1.00
**Health information from HEWs**				
No	15(12.5	105(87.5)	1.00	1.00
Yes	227(78.3)	63(21.7)	25.22(13.72, 46.36)	4.23(1.83, 9.70)*

*Variables with a *P* value of < 0.05 were considered statistically significant

^#^Peer friend, female classmates

## Discussion

In this study, the prevalence of good knowledge of obstetric danger signs was 59%; mothers’ educational status, monthly income, decision-making power on service utilization, and health information from HEWs were identified as independent predictors of obstetric danger signs.

In the current finding, prevalence of good knowledge on obstetric danger signs was consistent with the 55.1%, a study conducted elsewhere in Ethiopia [13]. However, the current finding was higher than that of a study conducted in Somali region, Ethiopia (15.5%) [11]. The observed discrepancy might be due to the residence. While 79.0% of the mothers in this study lived in urban areas, 79.7% (11) of the participants of the other study were rural dwellers. Mothers living in urban areas are more likely to have access to health services and information on obstetric danger signs. This might be a fertile ground for the increase of awareness on obstetric danger signs. Besides, the observed discrepancy might be due to the fact that the majority of mothers in the current study were more educated women (64.6%) compared to study conducted in Somali region, of which the majority of women were illiterate (94.8%) (11). Similarly, this study finding is higher compared with those of studies conducted in other developing countries, for example, South Africa (52%) [21] and Uganda (19%) [22]. The difference in the knowledge of women on danger signs between this study and studies in South Africa and Uganda (21, 22) might be due to the expansion of HEWs to rural and urban communities of Ethiopia and urban residence which might have created opportunities for raising the level of knowledge to some extent.

The odds of having good knowledge of obstetric danger signs were 6.86 times higher among mothers who had above secondary school education compared to mothers who had no formal education. This finding was similar to those of studies conducted in Uganda and elsewhere in Ethiopia [15, 22]. This might be due to the fact that education is important for increasing respondents’ level of understanding of health information. Educated women have better access to health service information and understand the health messages easily to utilize health services. In addition, educated women have greater confidence to make decisions to use health care services.

On the other hand, mothers who had more than 3500 ETB household monthly income were found to be more likely to have good knowledge compared to their counterparts. This result is in line with those of other local studies in Ethiopia [12, 13]. This may be explained by the fact that income plays an important part in enabling mothers to visit health institutions and seek health services, and there might be repeated visits that help women to expose information on obstetric danger signs. Income also increases access to health information through electronic devices (TV, radio, and mobile internet). Another significant variable, mothers who had health information on the danger signs of obstetric complications from HEWs were 4.23-fold more likely to be knowledgeable compared to mothers who did not have such information. This finding is similar to the result of a study conducted in rural Tanzania [17]. The probable reason might be that discussing health issues with health professionals is indispensable for getting clear and updated information regarding obstetric danger signs and HEWs have frequent contacts with women which could help to acquire knowledge on obstetric danger signs. However, mothers who decided on service utilization with their husbands were 86% times less likely to be knowledgeable compared to mothers who decided by themselves. This is because autonomy empowers mothers to take any action anytime on health-related matters. It is clear that mothers who have full autonomy to decide to seek care from reproductive and maternal health services are more likely to have enough information and knowledge on issues including the danger signs of pregnancy, labor and delivery, and postnatal period [16].

### Limitations of this study

This study showed the current level of knowledge on obstetric danger signs and its associated factors among postnatal clinic attending mothers in Bahir Dar, Ethiopia. However, the limitations of this study are stated as follows: the cross-sectional nature of the data had made it impossible to reach the causal relationship between the different independent variables and knowledge of women about obstetric danger signs. This study conducted was institution-based, so the finding of the study could not be generalized to the general population. Finally, the other limitation of this study relies in its cross-sectional design with knowledge evaluated after delivery, although having experienced potential obstetric complications can increase the awareness of danger signs. If one wants to decrease adverse health outcomes by adequate and timely obstetric management, knowledge should be assessed during pregnancy, before the onset of potential complications.

## Conclusion

In the study area, the overall knowledge on obstetric danger signs is poor. Thus, increasing mothers’ socio-economic status, educational level, and decision- making power on the utilization of health service are essential to mitigate the high burden of maternal morbidity due to obstetric complications. In addition, health information acquisition on the danger signs of obstetric complications from health professionals should be intensified.

## Data Availability

Authors present the data on the main paper.

## Abbreviations

ANC: Antenatal care
AOR: Adjusted odds ratio
CI: Confidence interval
COR: Crude odds ratio
EDHS: Ethiopian Demographic Health Survey
HEW: Health extension worker
MMR: Maternal mortality ratio
OR: Odds ratio
PNC: Postnatal care

## References

[1] Del Barco RC. Monitoring birth preparedness and complication readiness. Tools and indicators for maternal and newborn health. 2004.

[2] World Health Organization. Trends in maternal mortality: 1990 to 2008. Geneva: Estimates developed by WHO, UNICEF, UNFPA and The World Bank; 2010.

[3] World Health Organization (2014). United Nations agencies report steady progress in saving mothers’ lives.

[4] Requejo J, Victora C, Bryce J. Data resource profile: countdown to 2015: maternal, newborn and child survival. International Journal of Epidemiology. 2014;034. 10.1093/ije/dyu034 PMC3997378 24639449

[5] Hogan MC, Foreman KJ, Naghavi M, Ahn SY, Wang M, Makela SM, Lopez AD, Lozano R, Murray CJ (2010). Maternal mortality for 181 countries, 1980–2008: a systematic analysis of progress towards Millennium Development Goal 5. The Lancet..

[6] Central Statistical Authority [Ethiopia] and ORC Macro (2011). Ethiopia Demographic and Health Survey 2011.

[7] World Health Organization. The WHO application of ICD-10 to deaths during pregnancy, childbirth, and puerperium: ICD-MM. Geneva: World Health Organization; 2012.

[8] Family Care International, Maternal, and Newborn Health. Maryland, USA: Monitoring birth preparedness and complication readiness. Tools and indicators for maternal, and newborn health; 2004.

[9] The Federal Democratic Republic of Ethiopia, Ministry of Health: National Reproductive Strategy. Addis Ababa. Ethiopia: FMOH. 2006;2006:16–7..

[10] Starrs AM (2006). Safe motherhood initiative: 20 years and counting. The Lancet..

[11] Maseresha N, Woldemichael K, Dube L (2016). Knowledge of obstetric danger signs and associated factors among pregnant women in Erer district, Somali region Ethiopia. BMC Women's Health..

[12] Workineh Y, Hailu D, Gultie T, Degefu N, Mihrete M, Shimeles M, Mahino M, Guesh M, Alemu M (2014). Knowledge of obstetric danger signs and its associated factors in Arba Minch town Ethiopia. Am J Health Res..

[13] Amenu G, Mulaw Z, Seyoum T, Bayu H (2016). Knowledge about danger signs of obstetric complications and associated factors among postnatal mothers of Mechekel District Health Centers, East Gojjam Zone, Northwest Ethiopia, 2014. Scientifica.

[14] Hailu D, Berhe H (2014). Knowledge about obstetric danger signs and associated factors among mothers in Tsegedie District, Tigray Region, Ethiopia 2013: community-based cross-sectional study. Plos One..

[15] Bililign N, Mulatu T (2017). Knowledge of obstetric danger signs and associated factors among reproductive-age women in Raya Kobo district of Ethiopia: a community based cross-sectional study. BMC Pregnancy and Childbirth..

[16] Ossai EN, Uzochukwu BS (2015). Knowledge of danger signs of pregnancy among clients of maternal health service in urban and rural primary health centres of Southeast Nigeria. J Community Med Health Educ.

[17] Pembe AB, Urassa DP, Carlstedt A, Lindmark G, Nyström L, Darj E (2009). Rural Tanzanian women’s awareness of danger signs of obstetric complications. BMC Pregnancy and Childbirth..

[18] Federal Democratic Republic of Ethiopia Ministry of Health. Health Sector Transformation Plan. Ministry of health, 2015.

[19] Central Statistical Agency (2014). Ethiopia Mini Demographic and Health Survey, Addis Ababa, Ethiopia.

[20] Central Statistical Authority (2010). 2007 population and housing census of Ethiopia.

[21] Hoque M, Hoque ME (2011). Knowledge of danger signs for major obstetric complications among pregnant KwaZulu-Natal women: implications for health education. Asia Pac J Public Health.

[22] Kabakyenga JK, Östergren PO, Turyakira E, Pettersson KO (2011). Knowledge of obstetric danger signs and birth preparedness practices among women in rural Uganda. Reproductive Health..

